# Hypokalemic Periodic Paralysis: A Case Report of Acute Flaccid Quadriparesis Mimicking Guillain-Barré Syndrome

**DOI:** 10.7759/cureus.103769

**Published:** 2026-02-17

**Authors:** Lisha Michel M, Kiran Kumar

**Affiliations:** 1 Internal Medicine, Thumbay University Hospital, Ajman, ARE

**Keywords:** acute flaccid paralysis, electrolyte imbalance, guillain-barré syndrome, hypokalemia, periodic paralysis, quadriparesis

## Abstract

Hypokalemic periodic paralysis (HPP) is an uncommon but reversible cause of acute flaccid paralysis. It can clinically resemble neurological emergencies such as Guillain-Barré syndrome (GBS), leading to potential misdiagnosis and delays in appropriate treatment.

We report a 30-year-old man who presented with sudden-onset quadriparesis following an acute febrile illness with gastrointestinal symptoms characterized by fever, myalgia, arthralgia, and vomiting. Initial neurological assessment demonstrated proximal and distal weakness without sensory involvement. Laboratory tests revealed severe hypokalemia (2.2 mmol/L), mild hypomagnesemia, hypophosphatemia, and markedly elevated CRP and procalcitonin. An MRI of the brain and spine was unremarkable. Although GBS was considered, rapid improvement in muscle strength following intravenous (IV) and oral potassium supplementation supported the diagnosis of hypokalemic periodic paralysis. The patient made a full recovery with the correction of electrolytes.

This case emphasizes the importance of early electrolyte evaluation in acute flaccid paralysis and highlights the need to distinguish hypokalemic paralysis from GBS, as timely potassium replacement leads to rapid and complete recovery.

## Introduction

Hypokalemic periodic paralysis (HPP) is a rare neuromuscular disorder characterized by episodic muscle weakness associated with low serum potassium levels. It may be primary (familial) or secondary to conditions such as thyrotoxicosis, renal or gastrointestinal potassium losses, and viral illnesses [1]. Secondary forms are more common in adults and are often precipitated by infections, electrolyte losses, or endocrine disorders. HPP presents with acute flaccid weakness involving proximal more than distal muscles, typically without sensory deficits [2].

Its presentation may clinically mimic Guillain-Barré syndrome (GBS), a neurological emergency requiring prompt recognition. Misdiagnosis can lead to unnecessary investigations such as lumbar puncture, neurophysiological studies, or immunotherapy. Therefore, the early identification of hypokalemia is essential. Here, we report a case of severe hypokalemic paralysis masquerading as GBS and highlight the importance of this critical differential diagnosis [3].

## Case presentation

A 30-year-old man presented to the emergency department in a wheelchair with complaints of high-grade fever, severe myalgia, arthralgia, retro-orbital pain, throat pain, mild cough, nausea, and multiple episodes of vomiting since the previous day. He reported that around 3 AM on the day of presentation, he suddenly developed an inability to move both upper and lower limbs. There were no sensory complaints, dysphagia, urinary incontinence, or sphincter disturbances; however, he noted dark-colored urine.

On examination, the patient was febrile at 38.5°C, with a pulse of 74/minute, blood pressure of 114/65 mmHg, and respiratory rate of 20/minute; he appeared dehydrated, though cardiovascular, respiratory, and abdominal examinations were otherwise unremarkable. Neurological assessment revealed proximal and distal weakness in all four limbs, with upper limb power graded at 3/5 and lower limb power at 3/5 both proximally and distally. Lower limb reflexes were absent initially, while upper limb reflexes were preserved; plantar responses were downgoing, and sensory and cranial nerve examinations were normal. This constellation of acute quadriparesis with areflexia raised initial concern for Guillain-Barré syndrome (GBS).

Laboratory investigations demonstrated marked hypokalemia (2.2 mmol/L) with normal sodium and creatinine levels, mildly reduced bicarbonate (20.8 mmol/L), low phosphorus (1.7 mg/dL), and magnesium levels ranging from 1.94 to 1.97 mg/dL (Table 1). Inflammatory markers were elevated, including WBC counts of 26.3 × 10⁹/L, CRP from 160.1 mg/L, and procalcitonin at 28.27 ng/mL. Creatine phosphokinase (CPK) levels were moderately elevated (846 U/L). Vitamin B12 was notably low at 87 pg/mL, while viral serologies for influenza, mycoplasma, and COVID-19, as well as urine cultures, blood cultures, and thyroid function test, were negative.

**Table 1:** Initial Laboratory Parameters Severe hypokalemia, hypophosphatemia, B12 deficiency, elevated CRP/procalcitonin, elevated CPK, and hypomagnesemia CPK: creatine phosphokinase

Parameters	Patient Values	Reference Range
Serum potassium (K⁺)	2.2 mmol/L	3.5-5.0 mmol/L
Sodium (Na⁺)	139 mmol/L	135-145 mmol/L
Bicarbonate (HCO₃⁻)	20.8 mmol/L	22-29 mmol/L
Magnesium (Mg²⁺)	1.94-1.97 mg/dL	1.7-2.2 mg/dL
Phosphorus (PO₄³⁻)	1.7 mg/dL	2.5-4.5 mg/dL
Creatinine	Normal	0.6-1.3 mg/dL
WBC	26.3 → 15.9 → 11.9 × 10⁹/L	4-11 × 10⁹/L
CRP	160.1 → 55.7 mg/L	<5 mg/L
CPK	846-914 U/L	22-198 U/L
Procalcitonin	28.27 ng/mL	<0.05 ng/mL
Vitamin B12	87 pg/mL	200-900 pg/mL

MRI of the brain and spine and chest radiography were unremarkable, and arterial blood gas analysis showed a mild metabolic disturbance consistent with hypokalemia.

Management focused on the urgent correction of electrolyte abnormalities, including repeated cycles of intravenous (IV) potassium chloride (20 mmol in 500 mL normal saline over three hours), high-dose oral potassium supplementation, and concurrent magnesium and phosphorus replacement. Dextrose-containing fluids were avoided to prevent further intracellular potassium shifts, and the patient was encouraged to maintain good hydration and consume a potassium-rich diet.

Neurological status and serum electrolytes were monitored every four hours. As potassium levels improved from 2.2 to 3.8 to 4.4 mmol/L, a parallel rapid improvement in muscle strength was noted. Reflexes normalized, and no respiratory involvement occurred. Given these findings, the initial suspicion of GBS was dismissed (Tables 2, 3).

**Table 2:** Serum Potassium Correction

Time Points	Serum Potassium (mmol/L)	Clinical Correlation
During admission (zero hours)	2.2	Quadriparesis
~12-18 hours	3.8	Marked strength improvement
~24-36 hours	4.4	Near-complete recovery

**Table 3:** Neurological Examination With Reflex Status

Time After Treatment	Neurological Status
During admission (zero hours)	Upper limb, 3/5; lower limb, 3/5
12-24 hours	Strength improving; reflexes responding
24-48 hours	Upper limb, 5/5; lower limb, 4/5
Days 3-4	Ambulating without assistance

The patient also received IV antibiotics, ceftriaxone 2 g IV once daily, in view of fever, leukocytosis, and elevated inflammatory markers. His fever and inflammatory markers gradually improved over the next 24-48 hours.

By days 3-4 of treatment, the patient showed marked clinical improvement. Upper limb strength had fully recovered to 5/5, while lower limb power improved to 4-5/5, allowing him to ambulate with assistance. Vital signs stabilized, and inflammatory markers continued to decline in parallel with his neurological recovery. He was subsequently shifted to the ward and later discharged with symptomatic treatment and advised close follow-up.

## Discussion

Hypokalemic periodic paralysis (HPP) is an important reversible cause of acute flaccid paralysis and may closely resemble Guillain-Barré syndrome (GBS). Both conditions can present with sudden symmetrical weakness and depressed reflexes; however, HPP is distinguished by the presence of significant hypokalemia, normal sensory examination, and rapid recovery with potassium replacement. Because early electrophysiology in HPP may show markedly reduced compound motor action potential (CMAP) amplitudes, temporarily mimicking neuropathic patterns, misdiagnosis as GBS has been reported, particularly when electrolyte abnormalities are not promptly identified if serum electrolytes are not checked promptly [2-5].

Secondary HPP is often precipitated by infections, gastrointestinal losses, thyrotoxicosis, or medications. In this patient, a febrile illness with repeated vomiting likely triggered a severe drop in serum potassium. The associated elevations in CPK, along with mild hypophosphatemia and hypomagnesemia, reflect muscle fiber stress and may contribute to clinical weakness; the correction of all electrolytes is therefore essential. Rapid improvement in strength parallel to potassium normalization strongly supports HPP rather than GBS.

Early potassium testing in any patient with acute flaccid paralysis is critical, as failure to recognize HPP may lead to unnecessary lumbar puncture, nerve conduction studies, or intravenous immunoglobulin (IVIG) therapy [4-6]. With timely electrolyte correction and the management of the underlying trigger, prognosis is excellent, and recovery is typically rapid.

## Conclusions

Hypokalemic periodic paralysis should be considered in patients presenting with sudden flaccid quadriparesis, especially in the absence of sensory deficits. The early evaluation of serum electrolytes is essential to avoid misdiagnosis and initiate prompt treatment. This case reinforces the need for routine electrolyte assessment in acute flaccid paralysis, as the timely recognition of hypokalemia can prevent unnecessary investigations and ensure rapid recovery.
